# Perceived Ostomy odor and body image disturbance in colorectal cancer survivors: differences by ostomy status and sex

**DOI:** 10.1007/s00520-026-10566-5

**Published:** 2026-03-15

**Authors:** Juehyun Shin, Guofen Yan, Jessie S. Gibson, Randy A. Jones, Mikel Gray, Katrina J. Debnam

**Affiliations:** 1 School of Nursing, University of Virginia, Charlottesville, VA USA; 2 School of Medicine, University of Virginia, Charlottesville, VA USA

**Keywords:** Ostomy, Stoma, Colorectal cancer, Survivorship, Body image disturbance, Ostomy odor, Smell

## Abstract

**Purpose:**

Ostomy-related odor is a common, distressing symptom, yet its association with body image disturbance (BID) in colorectal cancer (CRC) survivors remains understudied. We examined whether perceived ostomy odor was associated with BID and whether this association varied by ostomy status (temporary vs. permanent) and sex.

**Methods:**

In a cross-sectional online survey of 130 CRC survivors with ostomies (Stages I–III), perceived ostomy odor was assessed with a single item and BID with the Body Image Scale. We used hierarchical multiple regression controlling for age, sex, BMI, cancer stage, time since ostomy surgery, and ostomy status, followed by interaction and stratified analyses.

**Results:**

Higher perceived ostomy odor was associated with greater BID (Model 2: *B* = 0.89, *p* = 0.006; Δ*R*^2^ = .044). The odor × ostomy status interaction was significant (*B* =  − 1.39, *p* = 0.031): odor was associated with BID among participants with a temporary ostomy (*B* = 1.98, *p* < 0.001) but not among those with a permanent ostomy (*B* = 0.21, *p* = 0.595). The odor × sex interaction was also significant (*B* =  − 1.44, *p* = 0.045): odor was associated with BID among women (*B* = 1.81, *p* = 0.003) but not men (*B* = 0.54, *p* = 0.133).

**Conclusion:**

Perceived ostomy odor was associated with greater BID, with stronger associations among participants with temporary ostomies and among women. Odor management may be a clinically relevant target for reducing BID in higher risk subgroups.

## Introduction

Body image disturbance (BID) is a common and clinically meaningful concern for colorectal cancer (CRC) survivors [1–3]. In a meta-analysis of CRC survivorship symptoms, BID affected 78.5% of individuals and ranked as the third most severe symptom among the 15 symptoms included in the pooled analysis, with particularly high concern among those with past or current ostomies [4]. Consistent with this pattern, among postoperative CRC patients, ostomy presence has been consistently associated with poorer body image, with the greatest risk reported by those with permanent ostomies [2, 3]. Prospective and longitudinal studies further suggest that body image may worsen over the months following ostomy surgery and may predict subsequent well-being [5–7]. In addition to ostomy-related factors, demographic and clinical characteristics may shape body image concerns and trajectories in oncology populations, including vulnerability among younger individuals and those with higher BMI [8] and potential influences of treatment intensity and late effects, for which cancer stage may serve as a proxy in CRC [3]. Worse BID has also been observed among women [1].

The conceptualization of BID in oncology has evolved to incorporate both visual and functional bodily changes, providing a more comprehensive framework [9–12]. Research has established that alterations in physical appearance and bodily functions significantly impact body image perception, as demonstrated in studies of head and neck cancer survivors experiencing eating and speech difficulties [11, 13]. However, when considering the full spectrum of sensory experiences, visual and functional aspects demonstrate profound interconnectivity rather than existing as separate entities, as functional changes to the body frequently manifest visible components. This interconnected nature of bodily perception extends beyond just visual and functional aspects to include other sensory modalities, particularly olfactory experiences in individuals with ostomies [14, 15]. Odor-related concerns in ostomy care are well positioned within multisensory frameworks of body image, because olfactory context can bias appearance- and self-evaluative judgments. In experimental settings, adding a pleasant fragrance (vs. clean air) increases ratings of self and other faces on attractiveness, confidence, and femininity, suggesting that olfactory cues can shift visual self- and other-perception [16]. Work on body odors similarly indicates that chemosensory cues can guide integrated person perception and social evaluation, even when visual information is limited [17].

Odor management is a common and emotionally charged challenge for people living with an ostomy [18, 19]. Odor functions not only as a physical stimulus but also as a socially interpreted signal that can threaten moral and cultural expectations, prompting ongoing “somatic work” to prevent embarrassment [20]. Consistent with this framing, quality-of-life and qualitative studies identify flatus and odor as persistent worries that can contribute to embarrassment, social withdrawal, and vigilance in public settings, even years after surgery [18, 19, 21]. Odor-related distress is also shaped by stigma and disgust processes: disgust responses to bodily waste cues can motivate avoidance and distancing and may amplify fears of odor detection [23]. In individuals with ostomies, bowel/colostomy-specific disgust sensitivity has been linked to greater perceived stigma and poorer adjustment, largely via stigmatization concerns [24]. These findings suggest that perceived odor can operate as a socially salient cue that reinforces self-consciousness and social withdrawal and may thereby contribute to negative self-perception [20].

The connection between ostomy odor and BID is further supported by research on cross-modal correspondences and multisensory integration [16, 25–27]. Olfactory cues can cross-modally influence person perception and evaluative judgments (e.g., attractiveness and other social appraisals), with effects often attributed to affective priming and learned contextual meaning [26–28]. Experimental evidence also suggests that fragranced body odor can shape social impressions (e.g., perceived confidence/self-esteem and attractiveness) [29]. More broadly, social–action frameworks propose that odor-related cues can interact with other sensory modalities (e.g., vision) in shaping perception–action links [30]. These lines of evidence provide a conceptual basis for examining perceived ostomy odor as a potentially consequential sensory cue for BID.

Despite these theoretical foundations and emerging empirical evidence, significant gaps remain in our understanding of odor-related body image concerns in cancer survivors with ostomies. Guided by Maurice Merleau-Ponty’s [31] view of the body as both object and subject, this study conceptualizes olfactory bodily changes as one component of overall sensory experience. Despite significant advances in olfaction research across neuroscience, psychology, and biotechnology, the application of these insights to medical contexts, especially in cancer survivors, remains underdeveloped. Current literature predominantly examines the positive effects of pleasant fragrances on body image, leaving a significant knowledge gap regarding the psychosocial impact of disease-related or treatment-related odors, such as those associated with ostomies. Moreover, existing ostomy research tends to examine visible bodily changes as the sole cause of BID, overlooking the cross-modal interaction between different senses, particularly between olfactory and visual perception. Furthermore, most studies have included participants with ostomies from various medical conditions, creating a heterogeneous sample that makes it difficult to separate ostomy-specific effects from those of underlying diseases. For example, BID may vary considerably between individuals with ostomies from inflammatory bowel disease versus CRC. This limitation has left gaps in our understanding of how ostomy-specific factors influence body image outcomes.

The present study examined the association between perceived ostomy odor and BID among CRC survivors living with an ostomy. We hypothesized that greater perceived odor would be associated with higher BID after accounting for key demographic and clinical characteristics. We also posited that the strength of this association may differ by ostomy status (temporary vs. permanent) and by sex, given clinical and social context differences that may shape odor-related distress and body image concerns.

## Methods

This cross-sectional study examined associations among ostomy odor and BID in CRC survivors living with an ostomy. We targeted ≥ 120 participants based on an a priori power analysis for multiple linear regression (*α* = 0.05; 80% power) to detect an incremental effect of perceived ostomy odor on BID (Δ*R*^2^ ≈ 0.05) beyond covariates. This sample also supported planned odor × ostomy status and odor × sex interaction tests while limiting model overfitting. Participants were recruited through online support networks affiliated with national ostomy and CRC organizations. Eligibility criteria included being 18 years or older, having a colostomy or ileostomy as a result of Stage I–III CRC, residing in the United States, and being able to complete an English-language survey. Individuals were excluded if they reported a pre-CRC diagnosis of a mood or anxiety disorder or had significant sensory impairments. The study was approved by the University of Virginia Institutional Review Board (Protocol No. 6816).

### Study procedures

Data collection occurred during August and September 2024 using an online survey platform (Qualtrics). Participants completed informed consent and a comprehensive questionnaire that included instruments measuring ostomy odor and BID, along with demographic and clinical information. To maintain data quality, we employed a validation strategy to detect potentially fraudulent responses, incorporating dynamic attention-check questions and consistency verifications. Participants who completed the survey successfully and met our validation standards were provided with electronic gift cards as incentives.

### Instruments

The survey collected demographic and clinical information including age, sex, marital status, race, cancer stage, tumor location, BMI, time elapsed since ostomy surgery, and ostomy status (temporary or permanent).

We assessed the severity of ostomy odor using a single item. Participants answered the question, “Do you experience embarrassing smells from your stoma bag?” This item employed a 7-point Likert scale (0 = “not at all” to 6 = “very much”), with higher scores indicating more severe issues. We selected a single-item approach because odor is a discrete, clinically salient symptom and is commonly captured in ostomy-specific clinical and quality-of-life tools as a stand-alone item (e.g., symptom checklists within broader ostomy function/impact instruments such as the Colostomy Impact (CI) score, which includes an odor item alongside other practical ostomy-related problems). In contrast to multi-item psychosocial constructs (e.g., BID), symptom-focused clinical measures may use brief items for feasibility; however, we acknowledge that a multi-item odor measure could better capture key dimensions of odor experience (e.g., frequency and situational triggers), as noted in “Limitations.”

BID was assessed using the Body Image Scale (BIS) [32]. The BIS is a 10-item self-report measure evaluating three dimensions of cancer-related body image: affective (e.g., feelings about femininity/masculinity and attractiveness), behavior (e.g., difficulty looking at oneself naked and avoiding social interactions due to appearance), and cognitive (e.g., satisfaction with appearance or scars). Participants rate each item on a four-point Likert scale (0 =  “not at all” to 3 =  “very much”), with total scores ranging from 0 to 30. Higher scores indicate greater BID. The BIS has been validated for use in various medical contexts, including colorectal disease [5, 7, 33]. It demonstrates high internal consistency (Cronbach’s *α* = 0.93) and good clinical validity and sensitivity to change. For this study, we modified the 10th item, replacing “your scar” with “your stoma” to better reflect the experiences of ostomy patients. In our study, the scale showed good internal consistency (Cronbach’s alpha = 0.88).

### Statistical analysis

To enhance data quality, we implemented data-cleaning procedures. This involved excluding cases with missing key demographic and clinical information (*n* = 5). We also screened continuous covariates for extreme values and removed outliers in age and BMI (*n* = 3) because these variables are established correlates of body image outcomes and, when extreme, can exert disproportionate leverage on regression estimates and assumption checks. We first used descriptive statistics to summarize participant characteristics. To examine associations between ostomy odor and BID, we conducted hierarchical multiple regression analyses. In Model 1, we entered demographic and clinical covariates (age, sex, BMI, cancer stage, time since ostomy surgery, and ostomy status). In Model 2, we added ostomy odor. To evaluate effect heterogeneity, we then tested moderation by adding an odor × ostomy status interaction (Model 3) and an odor × sex interaction (Model 4). To aid interpretation of significant interactions, we conducted stratified regression models by ostomy status and by sex. Statistical analyses were conducted using Jamovi 2.3.26 software [34]. Regression assumptions were evaluated using visual inspection of *Q*-*Q* plots and residual plots, along with collinearity diagnostics (tolerance and variance inflation factors) to evaluate multicollinearity.

## Results

Demographic and clinical characteristics of the participants (*n* = 130) are summarized in Table 1. The mean age was 44.3 years, and the mean BMI was 23.6 kg/m^2^. Most participants were male (67.7%), married or living with a partner (68.5%), and identified as White or Caucasian (66.2%). Stage II disease was the most common (60.0%), and tumor location was evenly distributed between the colon (48.5%) and rectum (46.9%). Slightly more than half of participants had permanent ostomies (53.8%), and most had undergone ostomy surgery within the past year (76.2%).

**Table 1:** Characteristics of participants (*n* = 130)

Characteristic		*n*	%
Sex	Female	42	32.3%
	Male	88	67.70%
Marital status	Married or living with a partner	89	68.50%
	Widowed, divorced, or separated	29	22.30%
	Never been married	12	9.2%
Race	White or Caucasian	86	66.2%
	Black or African American	29	22.30%
	Other races	15	11.5%
Cancer stage	Stage 1	31	23.8%
	Stage 2	78	60%
	Stage 3	21	16.2%
Cancer Location	Colon	63	48.5%
	Rectum	61	46.9%
	Both	6	4.6%
Ostomy Status	Temporary	60	46.2%
	Permanent	70	53.8%
Time since Ostomy	Less than 1 year	99	76.20%
	1 year or more	31	23.90%
**Characteristic**	**Range, mean (SD)**		
Age	22–67, 44.3 (10.7)		
BMI	16.8–30.4, 23.6 (2.85)		
**Main variables**	**Range, mean (SD)**		
Ostomy odor	0–6, 2.65 (1.47)		
Body image disturbance	4–29, 18.3 (5.87)		

As shown in Table 2, perceived ostomy odor is positively associated with BID after adjustment for demographic and clinical covariates. In the covariates-only model (Model 1), the predictors explained 27.3% of the variance in BID (*R*^2^ = 0.273). Men reported higher BID than women (*B* = 4.10, *p* < 0.001), and participants with a permanent ostomy reported higher BID than those with a temporary ostomy (*B* = 3.86, *p* < 0.001). Adding ostomy odor in Model 2 significantly improved model fit (Δ*R*^2^ = 0.044, *p* < 0.01; *R*^2^ = 0.317), with higher odor associated with higher BID (*B* = 0.89, *p* = 0.006). In Model 3, the odor × ostomy status interaction was significant (*B* =  − 1.39, *p* = 0.031; Δ*R*^2^ = 0.026; *R*^2^ = 0.343), indicating that the odor–BID association was stronger among participants with a temporary ostomy (*B* = 1.80, *p* < 0.001) than among those with a permanent ostomy (estimated *B* = 0.41). In Model 4, the odor × sex interaction was also significant (*B* =  − 1.44, *p* = 0.045; Δ*R*^2^ = 0.022; *R*^2^ = 0.365), indicating a stronger odor-BID association among women (*B* = 2.65, *p* < 0.001) than men (estimated *B* = 1.21).

**Table 2:** Hierarchical multiple regression models predicting BID from ostomy odor and interactions with ostomy status and sex

Predictors	Model 1 (covariates only)		Model 2 (+ odor)		Model 3 (+ odor × ostomy status)		Model 4 (+ odor × sex)	
	*B* (SE)	*p*	*B* (SE)	*p*	*B* (SE)	*p*	*B* (SE)	*p*
Intercept	8.30 (4.21)	0.051	5.40 (4.23)	0.204	3.30 (4.27)	0.441	1.66 (4.30)	0.699
Age (years)	0.04 (0.05)	0.379	0.03 (0.05)	0.562	0.03 (0.04)	0.468	0.04 (0.04)	0.404
Sex (male–female)	4.10 (1.02)	< 0.001***	3.46 (1.02)	< 0.001***	3.41 (1.00)	< 0.001***	6.80 (1.94)	< 0.001***
BMI	0.18 (0.17)	0.306	0.25 (0.17)	0.147	0.23 (0.17)	0.172	0.21 (0.16)	0.198
Cancer stage								
Stage 2–Stage 1	−0.96 (1.17)	0.412	−1.22 (1.14)	0.286	−1.52 (1.13)	0.18	−1.36 (1.12)	0.225
Stage 3–Stage 1	−1.80 (1.49)	0.23	−2.57 (1.48)	0.085	−2.75 (1.46)	0.062	−2.73 (1.44)	0.061
Time since ostomy surgery (1 year or more )-(less than 1 year)	−0.03 (1.11)	0.977	0.21 (1.09)	0.845	0.32 (1.07)	0.764	−0.07 (1.08)	0.95
Ostomy status (permanent-temporary)	3.86 (0.97)	< 0.001***	4.21 (0.95)	< 0.001***	7.86 (1.92)	< 0.001***	7.14 (1.92)	< 0.001***
Odor			0.89 (0.32)	0.006**	1.80 (0.52)	< 0.001***	2.65 (0.67)	< 0.001***
Odor x ostomy Status odor x (permanent-temporary)					−1.39 (0.63)	0.031*	−1.07 (0.64)	0.1
Odor x sex Odor x (male–female)							−1.44 (0.71)	0.045*
*R*^2^	0.273		0.317		0.343		0.365	
Δ*R*^2^			0.044**		0.026*		0.022*	

Values are unstandardized regression coefficients (*B*) with standard errors (SE). For categorical predictors, the second group listed is the reference category (e.g., male–female indicates female is the reference). Δ*R*^2^ represents the increase in explained variance compared to the previous model. * *p* <.05; ** *p* <.01; and *** *p* <.001

As shown in Table 3, the stratified models further clarify the interaction patterns observed in Table 2. When stratified by ostomy status, perceived ostomy odor was strongly associated with higher BID among participants with a temporary ostomy (*B* = 1.98, *p* < 0.001), whereas odor was not significantly associated with BID among those with a permanent ostomy (*B* = 0.21, *p* = 0.595). Within the permanent ostomy subgroup, sex remained a significant correlate of BID (*B* = 6.73, *p* < 0.001). When stratified by sex, perceived ostomy odor was significantly associated with higher BID among women (*B* = 1.81, *p* = 0.003) but not among men (*B = *0.54, *p* = 0.133). Among men, permanent (vs. temporary) ostomy status was associated with higher BID (*B* = 6.09, *p* < 0.001). Overall, these subgroup-specific estimates indicate that the odor–BID association was most pronounced among participants with a temporary ostomy and among women, whereas BID among men was more strongly related to ostomy permanence.

**Table 3:** Regression models predicting BID, stratified

Stratified by ostomy status				
Predictors	Temporary ostomy		Permanent ostomy	
	*B* (SE)	*p*	*B* (SE)	*p*
Intercept	12.50 (4.85)	0.013*	3.40 (6.17)	0.584
Age (years)	−0.03 (0.05)	0.555	0.10 (0.07)	0.166
Sex (male–female)	−0.46 (1.19)	0.701	6.73 (1.52)	< 0.001***
BMI	−0.01 (0.19)	0.968	0.35 (0.26)	0.181
Cancer stage				
Stage 2–Stage 1	−0.44 (1.51)	0.772	−1.41 (1.52)	0.355
Stage 3–Stage 1	−0.05 (2.04)	0.98	−2.96 (1.91)	0.127
Time since ostomy surgery (1 year or more)-(less than 1 year)	0.41 (1.69)	0.808	0.76 (1.33)	0.57
Odor	1.98 (0.46)	< 0.001***	0.21 (0.39)	0.595
**Stratified by sex**				
**Predictors**	**Female**		**Male**	
	B (SE)	*p*	B (SE)	*p*
Intercept	8.10 (6.51)	0.22	11.24 (5.12)	0.031*
Age (years)	0.02 (0.07)	0.756	0.02 (0.05)	0.749
BMI	0.07 (0.27)	0.789	0.17 (0.20)	0.395
Cancer stage				
Stage 2–Stage 1	0.85 (1.87)	0.652	−1.39 (1.35)	0.307
Stage 3–Stage 1	2.15 (2.16)	0.327	−4.33 (1.96)	0.03*
Time since ostomy surgery (1 year or more)-(less than 1 year)	−2.40 (1.85)	0.202	0.38 (1.31)	0.775
Ostomy status (permanent-temporary)	−0.28 (1.62)	0.864	6.09 (1.10)	< 0.001***
Odor	1.81 (0.57)	0.003**	0.54 (0.36)	0.133

Values are unstandardized regression coefficients (*B*) with standard errors (SE). For categorical predictors, the second group listed is the reference category. * *p* <.05; ** *p* <.01; and *** *p* <.001

## Discussion

In this study of colorectal cancer survivors living with ostomies (*n* = 130), perceived ostomy odor was positively associated with BID after adjustment for demographic and clinical characteristics. Evidence of effect heterogeneity was observed, with significant odor × ostomy status and odor × sex interactions in the full-sample models. In stratified analyses, perceived odor was strongly associated with higher BID among participants with a temporary ostomy but not among those with a permanent ostomy, and among women but not among men. These findings extend prior work linking ostomy-related experiences to body image outcomes by highlighting perceived odor as a distinct, clinically relevant contributor to BID in specific subgroups. Building on the multisensory rationale outlined in the Introduction, the present results underscore a clinically salient point: subjective odor experience may be consequential for body image even when objective detectability is uncertain. This interpretation aligns with evidence that odor-related effects on appraisal can be shaped by learned meaning and expectations [25, 26].

Across the full-sample models in Table 2, men report higher BID than women, whereas the broader CRC survivorship literature often reports comparable or greater body image concerns among women [1]. In contrast, the finding that permanent (vs. temporary) ostomy status was associated with higher BID is broadly consistent with evidence that permanent ostomies can be linked to poorer body image and greater adjustment burden [2, 3]. The interaction and stratified results further suggest that perceived odor is more strongly related to BID among women and among participants with a temporary ostomy. These patterns may reflect differences in the social meaning and day-to-day salience of odor and other ostomy-related bodily cues. For example, temporary ostomies may involve greater uncertainty and transitional expectations that heighten attention to sensory concerns, whereas permanent ostomies may concentrate adjustment demands around longer-term functional and identity-related implications of living with an ostomy [2, 3, 24].

Consistent with survivorship and qualitative work, odor-related distress may also be shaped by ongoing monitoring and anticipatory concern in social settings. Worries about flatus and odor are often described as difficult to fully control and socially consequential, contributing to vigilance and self-consciousness in everyday contexts [18, 19]. In this context, perceived odor may contribute to BID through both direct sensory experience and anticipation of negative evaluation [35]. Disgust-related and stigma-related processes may further exacerbate odor-related body image concerns. Bodily waste cues can elicit disgust and avoidance [22]. While these mechanisms were not directly tested here, they represent plausible psychosocial pathways through which perceived odor could amplify self-consciousness and social withdrawal, thereby sustaining BID.

From a clinical perspective, perceived odor may represent a modifiable target for reducing BID in higher risk subgroups. Ostomy care guidance highlights low-burden strategies (e.g., dietary experimentation, deodorants during appliance emptying, and charcoal flatus filters) that may reduce odor-related distress and support confidence in daily activities [19]. Randomized trials further suggest that odor-focused approaches (e.g., essential oils placed in the stoma bag) can reduce perceived odor and improve patient-reported outcomes relevant to adjustment and well-being [15, 21]. In addition to practical management, assessment of perceived stigma and disgust-related distress may help identify individuals who could benefit from targeted psychosocial support alongside ostomy self-management education [23].

### Strengths and limitations

This study addresses an understudied aspect of cancer survivorship by examining perceived ostomy odor as a potential predictor of BID; to our knowledge, it is among the first studies to empirically evaluate the odor-body image link in an oncology population, helping extend multisensory and stigma-related frameworks of body image to clinically relevant, symptom-based olfactory experiences. However, several limitations should be noted when interpreting these findings, including the cross-sectional design, the use of a single self-report item to assess odor (which may not capture key dimensions such as frequency, situational triggers, or discrepancies between perceived vs. detectable odor), and recruitment from online support networks with limited demographic diversity (which may limit generalizability).

### Future research recommendations

Future studies should use longitudinal designs to examine how odor-related distress and BID evolve across the trajectory of ostomy adaptation, including key clinical transition points. Research should also validate more comprehensive measures of odor-related experiences and incorporate objective indicators where feasible. Finally, intervention studies are needed to test whether improving odor management reduces BID and whether effects differ by ostomy status or sex.

## Data Availability

The data that support the findings of this study are available from the corresponding author upon reasonable request.
